# A Comprehensive dataset for Australian mine production 1799 to 2021

**DOI:** 10.1038/s41597-023-02275-z

**Published:** 2023-06-20

**Authors:** Gavin M. Mudd

**Affiliations:** Environmental Engineering, School of Engineering, RMIT University, 124 La Trobe Street, Melbourne, VIC 3000 Australia

**Keywords:** Economic geology, Environmental impact

## Abstract

Given that metals, minerals and energy resources extracted through mining are fundamental to human society, it follows that accurate data describing mine production are equally important. Although there are often national statistical sources, this typically includes data for metals (e.g., gold), minerals (e.g., iron ore) or energy resources (e.g., coal). No such study has ever compiled a national mine production data set which includes basic mining data such as ore processed, grades, extracted products (e.g., metals, concentrates, saleable ore) and waste rock. These data are crucial for geological assessments of mineable resources, environmental impacts, material flows (including losses during mining, smelting-refining, use and disposal or recycling) as well as facilitating more quantitative assessments of critical mineral potential (including possible extraction from tailings and/or waste rock left by mining). This data set achieves these needs for Australia, providing a world-first and comprehensive review of a national mining industry and an exemplar of what can be achieved for other countries with mining industry sectors.

## Background & Summary

Hewett (1929)^1^ neatly articulates the historical evolution of the mining industry reflecting the progression of human society’s needs, demands and population size. That is, the range of metals, minerals and energy resources mined have increased over time as well as the quantity extracted annually from the earth (e.g., Sykes *et al*.^2^; Greenfield *et al*.^3^). After all, various ages of civilization are known by certain metals or minerals – the Stone, Bronze, Iron and Atomic Ages. Given this evolution, it is therefore important to synthesize accurate data sets which help illustrate this history, thereby providing a foundation to help explore the many issues and questions which arise when assessing mining: its history, scale, impacts (social and environmental), economics, the role of geology and mineral exploration and especially the question of scarcity or depletion of mineable resources.

The process of mining can be simplified to the following: mineral exploration finds a notable deposit of economic potential, approvals for mining are sought and obtained, the mine is built and begins operations, the deposit is eventually depleted or becomes uneconomic and the mine is closed and the site rehabilitated, and the cycle begins again (e.g., Spitz & Trudinger^4^; Darling^5^; IIED & WBCSD^6^). Mining operations can be conducted through open pit or underground techniques, producing ‘ore’ which contains economic concentrations of the target metals or minerals as well as ‘waste rock’ with low to uneconomic concentrations of metals or minerals. The ore is processed to produce a saleable product such as a metal-rich concentrate (e.g., copper, nickel, lead, zinc or tin-rich concentrates), beneficiated mineral concentrate (e.g., saleable iron ore, beneficiated bauxite, washed coal) or metallic product (e.g., gold-silver doré bars, copper metal). After removal of the saleable product, the remaining minerals are called ‘tailings’ and are typically discharged to an engineered storage dam while waste rock is usually placed in large piles.

From this basic review, there are already important data which arise – the size of a mineral deposit (in tonnes), the grades of metals or minerals to be extracted (say per cent or grams per tonne) as well as cumulative ore processed, grades, production, tailings and waste rock. At a national (or even global) level, the sum of all mines gives total production an annual basis or this data can be summed to arrive at cumulative production over time. It has been common since the late 1800s for state/provincial and national level governments to compile statistics on mining, the most prominent being tables of annual metals and minerals produced by mining. Some reporting included a detailed review of mining operations themselves, including aspects such as ore processed, ore grades (though not always) and products extracted, but such data has never been synthesized over time to build a unified data set of production at a national scale on a mine-by-mine or field-by-field basis.

There are numerous strong reasons that such data sets are crucial to develop:

- *Resource Scarcity*/*Depletion* – given that a mineral deposit is a finite quantity, concerns are regularly raised about the risks of mineral resource depletion^7,8^. In other words, are we at risk of ‘running out’ of resources? Examining trends in mining over time are fundamental to help address this concern.
- *Mineral Exploration and Geology* – trends in mining production can help in understanding the different mineral deposit types which have been mined over time^9^, which in turn can inform mineral exploration and provide a sound basis for assessing future potential mine supply of metals, minerals and energy resources.
- *Environmental and Social Impacts* – mining brings significant environmental and social risks, especially as the modern scale of mining is vastly larger than much older generations of mines. A detailed data set allows exploration of the myriad of complex questions and issues which relate to environmental and social impacts and benefits. For example, the effects of workers’ strikes or war on production levels (e.g., lead in Australia^10^), changing socioeconomic expectations^11^ or the increasing scale of mining leading to greater environmental risks to manage with modern mines (especially tailings, acid and metalliferous drainage, water resources, greenhouse gas pollution, etc)^4,6,12,13^.
- *Material Flows and Industrial Ecology* – it is important to be able to quantify and model the flows of metals, minerals, energy and materials through society’s use (aka industrial ecology). Given that metals (and sometimes minerals) can be readily recycled, often with a much lower environmental impact, this makes it important to understand the quantities residing in urban and industrial stocks, flows between sectors of the economy (including recycling) as well as those in mineral resources in order that policy settings optimise the availability of metals and minerals but with the lowest environmental and social impacts^14,15^.
- *Critical Minerals* – certain metals and minerals are widely considered to be so important to the trajectory of modern technology and societal goals but are vulnerable to supply disruptions that they are classified as ‘critical minerals’ (e.g., cobalt, rare earths, indium, tellurium)^16,17^. Many of the critical minerals are not mined in their own right, however, but remain as by-products at smelters and refineries (e.g., indium is recovered at zinc refineries), meaning that it is important to understand the host metals in order to understand that critical mineral^17,18^.

This data set was synthesized to provide the foundation for much of the above. It is a carefully constructed body of data covering cumulative individual mining project and field production to 2021 in Australia and includes detailed time series to validate the coverage of the mine-by-mine and field-by-field data. No such data set has ever been published on a national basis, let alone for a major global mining country like Australia. The data set provides a template for such a synthesis for other major mining nations. The data should prove highly valuable for a range of purposes and users alike.

## Methods

The basic approach adopted to synthesize these data are to compile mine production data on a field or individual mining project basis. The aspects included are the ore processed (tonnes), ore grades (g/t, %), recovered products (e.g., concentrates, metals, minerals, saleable ore), waste rock and contained metals and/or minerals extracted. All data are converted to metric units (based on the gram of weight), although the tonne is used and represents one million grams (i.e., 1 tonne = 1 Mg = 10^6^ g). The data are presented as cumulative totals to the end of the year 2021. For some sites with partial data, best estimates are used to fill the gaps based on other directly reported data (e.g., ore grades are missing in some years but can be estimated based on typical recovery rates or assumed from reserves grades).

In order to check the extent to which individual field and mine data cover national production, time series data are also included, representing cumulative Australia mine production for that metal or mineral. Herein, primary data sources and references by each individual state or territory, field and mine are described, although for mines only the general process is described (not the complete list of companies, as this can be immense over the two centuries which this data covers – such as the hundreds of companies operating around the Bendigo gold field since 1851 alone; besides, the collective data for such fields is captured by state statistics).

A more detailed list of state and federal government department and agencies over time is provided in Supplementary Information, including current names and website links.

Principal References – Australia

- Kalix *et al*., 1966, *Australian Mineral Industry: Production and Trade, 1842–1964*^19^.
- *BMR Annual Mineral Industry Review*, Years 1948 to 1987 (annual series)^20^.
- *ABARE Australian Mineral Statistics*, Years 1988 to 2011 (quarterly journal)^21^.
- *ABARE Australian Commodity Statistics*, Years 1986 to 2010 (including formerly Commodity Statistical Bulletin; annual series)^22^.
- *OCE Resources and Energy Statistics*, Years 2011 to 2022 (quarterly series)^23^.

Principal References – Tasmania:

- *TDM Annual Report*, Years 1882 to 1991/92 (annual series)^24^.
- *MRT Annual Review*, Years 1992/93 to 2010/11 (annual series)^25^.

Principal References – Victoria:

- Brough Smyth, 1869, *The Gold Fields and Mineral Districts of Victoria With Notes on the Modes of Occurrence of Gold and Other Metals and Minerals*^26^.
- *VDM Annual Report*, Years 1870 to 2007 (annual series)^27^.
- *VDM Gold Fields Statistics*, Years 1860 to 1918 (annual & quarterly series)^27^.
- *VDM Gold and Mineral Statistics*, Years 1919 to 1949 (annual series)^28^.
- *VDM Statistics Relating to the Mining Industry*, Years 1950 to 1976 (annual series)^29^.
- *VDPI Statistical Review*, Years 1997/98 to 2020/21 (annual series)^30^.

Principal References - New South Wales:

- *NSWDM Annual Report*, Years 1875 to 1989/90 (annual series)^31^.
- *NSWDMR Mineral Industry Review*, Years 1980 to 2010 (annual series)^32^.
- Miscellaneous minerals production statistics published online (e.g., Department websites).

Principal References - Queensland:

- *QDM Annual Report*, Years 1877 to 1991/92 (annual series)^33^.
- *QDNRME Queensland Minerals and Petroleum Review*, Years 1989 to 2008 (annual series)^34^.
- *QDNRM Queensland Metalliferous and Industrial Minerals*, Years 2011 to 2016 (annual series)^35^.
- Miscellaneous minerals production statistics published online (e.g., Department websites).

Principal References - South Australia:

- *SADM Mining Review: A Short Review of Mining Operations in the State of South Australia*, Half-Years Dec. 1903 to Dec. 1988 (6-monthly series)^36^.
- *SADME Mineral Production Statistics*, Half-Years Dec. 1978 to Dec. 2021 (6-monthly series)^37^.

Principal References - Northern Territory:

- *DNT Annual Report of the Administrators of the Northern Territory*, Years 1911 to 1969/70 (annual series)^38^.
- Balfour, 1989, *Administrator’s Reports for the Warramunga Gold Field (Tennant Creek) 1924–1969*^39^.
- Balfour, I S (Editor), 1990, *Government Resident’s Reports – The Top End Goldfields (Agicondi, Waggaman, Daly River & Katherine) 1870–1910*^40^.
- Balfour, I S (Editor), 1991, *Administrator’s Reports – The Top End Goldfields (Agicondi, Waggaman, Daly River & Katherine) 1911–1969*^41^.
- Ahmad, M & Munson, T J (Editors), 2013, *Geology and Mineral Resources of the Northern Territory*^42^.

Principal References - Western Australia:

- *WADM Annual Report*, Years 1894 to 2020/21 (annual series)^43^.
- *WADM Statistics Digest*, Years 1984 to 2020/21 (6-monthly series, now annual)^44^.

Miscellaneous References or Sources:

- Company reports to the Australian Securities Exchange (ASX) (quarterly, annual, media releases) and websites^45^.
- Technical Reports (environmental impact statements, mineral resource reports, feasibility studies, etc).
- Scientific papers, technical monographs (e.g., bulletins, research reports, Australasian Institute of Mining and Metallurgy monographs, books and conference proceedings), textbooks, academic theses, conference presentations (e.g.^46–52^, amongst many others).
- *The Mineral Industry: Its Statistics, Technology and Trade*^53^.
- *USBoM Minerals Yearbook*, Years 1932 to 1993^54^.
- *USBoM Commodity Data Summaries*, Years 1957 to 1977^55^ and *Mineral Commodity Summaries*, Years 1978 to 1995^56^.
- *USGS Minerals Yearbook*, Volumes 1 Metals and Minerals^57^ and 3 International^58^, Years 1994 to 2018 (including advance data releases to 2021).
- *USGS Mineral Commodity Summaries*, Years 1996 to 2023^59^.
- Mudd, G M, 2009, *The Sustainability of Mining in Australia: Key Production Trends and Their Environmental Implications for the Future*^60^.
- *RIU Register of Australian Mining*, Years 1978 to 2006 (annual series)^61^.
- *LP & Minmet The Australian Mines Handbook*, Years 1978 to 2005 (annual series)^62^.
- Specific industry organisations, such as the *World Nuclear Association* for uranium^63^.

Specific Notes on Each Metal/Mineral:

- Gold (Au) – contained Au in metallic form, doré bars, concentrates (Au, Cu or mixed) or direct shipping ore.
  - *Mass Units* – historically Au was reported in imperial units of fine troy ounces (oz), pennyweights (dwt) and grains (gr), with 24 grains per pennyweight, 20 penny weights per ounce. For this study, 1 ounce = 31.1 grams. All values converted to metric kilograms.
  - *Grade Units* – all converted to grams per metric tonne (g/t Au).
  - Conversions 1 oz Au = 20 dwt, 1 dwt = 24 gr or 1 oz = 480 gr.
  - Conversions 1 gram Au = 0.03215 g Au or 1 oz Au = 31.1 g.

- Silver (Ag) – contained Ag in metallic form, doré bars, concentrates (Au, Cu, Pb-Zn or Ni) or direct shipping ore.
  - *Mass Units* – historically Ag was reported in imperial units of fine troy ounces (oz), pennyweights (dwt) and grains (gr), with 24 grains per pennyweight, 20 penny weights per ounce. For this study, 1 ounce = 31.1 grams. All values converted to metric kilograms.
  - *Grade Units* – all converted to grams per metric tonne (g/t Ag).
- Bauxite – typically reported as in either raw form (e.g., Darling Ranges mines) or beneficiated saleable bauxite (e.g., Weipa).
  - *Mass Units* – dry metric tonnes (sometimes reported as wet metric tonnes and converted using moisture content; if no moisture reported, 5% is typically assumed).
  - *Grade Units* – typically includes percent alumina (%Al_2_O_3_).
- Alumina – reported as saleable smelter grade alumina and alumina chemicals.
  - *Mass Units* – dry metric tonnes.
  - *Grade Units* – may include percent alumina (%Al_2_O_3_, noting that manufactured alumina is a high purity mineral in any case).
- Black Coal – reported as raw coal or saleable coal.
  - *Mass Units* – dry metric tonnes (adjusted for moisture content if reported as wet tonnes).
  - *Grade Units* – typically energy content (e.g., GJ/t) as well as impurities such as silica (SiO_2_), alumina (Al_2_O_3_) or others.
- Brown Coal – reported as raw coal.
  - *Mass Units* – dry metric tonnes (adjusted for moisture content if reported as wet tonnes).
  - *Grade Units* – typically energy content (e.g., GJ/t).
- Cobalt (Co) – contained Co in metallic form, concentrates (Co, Ni, Cu or Pb-Zn) or direct shipping ore.
  - *Mass Units* – dry metric tonnes (adjusted for moisture content if reported as wet tonnes).
  - *Grade Units* – all converted to either percent (%Co) or grams per metric tonne (g/t).
- Copper (Cu) – contained Co in metallic form, concentrates (Cu, Ni, Pb-Zn or Sn) or direct shipping ore.
  - *Mass Units* – dry metric tonnes (adjusted for moisture content if reported as wet tonnes).
  - *Grade Units* – all converted to either percent (%Cu) or grams per metric tonne (g/t).
- Diamonds – contained diamonds in raw mineral form or direct shipping ore.
  - *Mass Units* – the carat has been maintained due its widespread use in the diamond sector, where 1 carat = 0.2 g.
  - *Grade Units* – all converted to carats per metric tonne (carats/t).
- Iron (Fe) Ore – typically reported as raw form (e.g., Middleback Ranges mines), beneficiated saleable iron ore (e.g., most Pilbara mines) or as iron ore concentrate (e.g., magnetite mines such as Savage River or Sino-Cape Preston).
  - *Mass Units* – dry metric tonnes (sometimes reported as wet metric tonnes and converted using moisture content; if no moisture reported, 5% is typically assumed).
  - *Grade Units* – typically includes percent iron (%Fe) and a range of impurities such as silica (%SiO_2_), alumina (%Al_2_O_3_), vanadium (%V), chromium (%Cr), phosphorous (%P), sulfur (%S), etc.
- Manganese (Mn) – typically reported as raw form (if sufficiently high grade), beneficiated saleable Mn ore or as Mn concentrate.
  - *Mass Units* – dry metric tonnes (sometimes reported as wet metric tonnes and converted using moisture content; if no moisture reported, 5% is typically assumed).
  - *Grade Units* – typically includes percent manganese (%Mn) or as manganese oxide (%MnO_2_), with the latter being the Mn mineral pyrolusite.
  - Conversions 1 tonne Mn = 1.582 t MnO_2_ or 1 t MnO_2_ = 0.632 t Mn.
- Nickel (Ni) – contained Ni in metallic form, concentrates (primarily Ni-dominant only) or direct shipping ore.
  - *Mass Units* – dry metric tonnes (adjusted for moisture content of reported as wet tonnes).
  - *Grade Units* – all converted to percent (%Ni).
- Lead (Pb) – contained Pb in metallic form, concentrates (Pb, Zn, Cu or mixed) or direct shipping ore.
  - *Mass Units* – dry metric tonnes (adjusted for moisture content if reported as wet tonnes).
  - *Grade Units* – all converted to percent (%Pb).
- Zinc (Zn) – contained Zn in metallic form, concentrates (Zn, Pb, Cu or mixed) or direct shipping ore.
  - *Mass Units* – dry metric tonnes (adjusted for moisture content if reported as wet tonnes).
  - *Grade Units* – all converted to percent (%Zn).
- Tin (Sn) – contained Sn in metallic form, tin oxides (e.g., as cassiterite extracted through alluvial prospecting and mining), concentrates (Sn, Cu or mixed) or direct shipping ore.
  - *Mass Units* – dry metric tonnes (adjusted for moisture content if reported as wet tonnes).
  - *Grade Units* – all converted to percent (%Sn).
- Uranium (U) – contained U in oxide concentrates or direct shipping ore, typically reported as uranium oxide (U_3_O_8_).
  - *Mass Units* – dry metric tonnes (adjusted for moisture content if reported as wet tonnes).
  - *Grade Units* – all converted to percent oxide (%U_3_O_8_), noting the conversion 1 tonne U = 1.179 t U_3_O_8_.
  - Conversions 1 tonne U = 1.179 t U_3_O_8_ or 1 t U_3_O_8_ = 0.848 t U.
- Gallium (Ga) – contained Ga in metallic form, oxides, concentrates or direct shipping ore.
  - *Mass Units* – dry metric tonnes (adjusted for moisture content if reported as wet tonnes).
  - *Grade Units* – all converted to either percent (%) or grams per metric tonne (g/t).
- Vanadium (V) – contained V in metallic form, oxides, concentrates or direct shipping ore.
  - *Mass Units* – dry metric tonnes (adjusted for moisture content if reported as wet tonnes).
  - *Grade Units* – all converted to either percent (%V), noting the conversion 1 tonne V = 1.785 t V_2_O_5_.
  - Conversions 1 tonne V = 1.785 t V_2_O_5_ or 1 t V_2_O_5_ = 0.0.560 t V.
- Antimony (Sb) – contained Sb in metallic form, oxides, concentrates (e.g., Sb-Au, Pb) or direct shipping ore.
  - *Mass Units* – dry metric tonnes (adjusted for moisture content if reported as wet tonnes).
  - *Grade Units* – all converted to either percent (%) or grams per metric tonne (g/t).
- Chromium (Cr) – contained Cr in metallic form, oxides, concentrates or direct shipping ore.
  - *Mass Units* – dry metric tonnes (adjusted for moisture content if reported as wet tonnes).
  - *Grade Units* – all converted to percent (%Cr_2_O_3_).
  - Conversions 1 tonne Cr = 1.462 t Cr_2_O_3_ or 1 t Cr_2_O_3_ = 0.684 t Cr.
- Graphite – contained graphite in mineral form or direct shipping ore.
  - *Mass Units* – dry metric tonnes (adjusted for moisture content if reported as wet tonnes).
  - *Grade Units* – all converted to percent total graphitic carbon (%TGC) where needed.
- Lithium (Li) – contained Li in metallic form, oxides, concentrates (e.g., spodumene mineral concentrate) or direct shipping ore.
  - *Mass Units* – dry metric tonnes (adjusted for moisture content if reported as wet tonnes).
  - *Grade Units* – all converted to percent (%Li) or as oxide (%Li_2_O).
  - Conversions 1 tonne Li = 2.153 t Li_2_O or 1 t Li_2_O = 0.465 t Li.
- Molybdenum (Mo) – contained Mo in concentrates (e.g., molybdenite concentrate, typically >85% MoS_2_) or direct shipping ore.
  - *Mass Units* – dry metric tonnes (adjusted for moisture content if reported as wet tonnes).
  - *Grade Units* – all converted to percent (%Mo).
  - Conversions 1 tonne Mo = 1.669 t MoS_2_ or 1 t MoS_2_ = 0.599 t Mo.
- Phosphate Rock – phosphate rock graphite in raw form (direct shipping ore) or beneficiated.
  - *Mass Units* – dry metric tonnes (adjusted for moisture content if reported as wet tonnes).
  - *Grade Units* – may include percent phosphate (%P_2_O_5_).
- Platinum Group Elements (PGEs) – this includes platinum (Pt), palladium (Pd), rhodium (Rh), ruthenium (Ru), osmium (Os) and iridium (Ir), typically extracted as placer Pt, placer ‘osmiridium’ (a common term for Os-Ir rich placers), contained in Ni concentrates or direct shipping ore.
  - *Mass Units* – internationally, PGEs are typically reported in imperial units of fine troy ounces (oz) although metric units are becoming more common.
  - *Grade Units* – all converted to grams per metric tonne (g/t).
  - Placer Pt – nuggets found in some NSW streams, with an average composition of 75.9% Pt, 0.13% Rh, 1.17% Ru and 1.3% Ir^53^.
  - Placer ‘Osmiridium’– due to their fundamental difference to placer Pt, osmiridium was the common name used for Os-Ir rich nuggets found in some VIC and TAS streams, with an average composition of 58.1% Ir, 33.5% Os, 2.7% Ru and 0.30% Rh^53^.
- Rare Earth Elements (REEs) – this includes the lanthanide series of elements from lanthanum (La) to ytterbium (Yb), typically extracted as mineral concentrates (e.g., monazite concentrates) or direct shipping ore.
  - *Mass Units* – dry metric tonnes (adjusted for moisture content if reported as wet tonnes).
  - *Grade Units* – all converted to percent elements (%REE) or oxides (%REO).
  - Conversions – converting between elemental and rare earth oxides is complex, as the elements are always found combined or mixed together. Total rare earth oxides only are given in this study. From 1942 to 2002, rare earths are assumed as extracted from monazite (and minor xenotime) concentrates produced from heavy mineral sands mining, adopting an average content of 60% REO in monazite and xenotime.
  - Conversions – based on global average REE grades, 1 t REO = 0.8304 t REE or 1 t REE = 1.2043 REO.
- Tungsten (W) – usually found in minerals from the wolframite group (X.WO_4_, where X can be Fe, Mn or others) or scheelite (CaWO_4_), often mined in alluvial or hard rock settings.
  - *Mass Units* – dry metric tonnes (adjusted for moisture content if reported as wet tonnes).
  - *Grade Units* – all converted to percent elements (%W) or most commonly as oxides (%WO_3_).
  - Conversions – assuming ferberite (FeWO_4_) as the principal wolframite mineral produced gives 1 t wolfram = 0.605 t W or 1 t W = 1.652 t wolfram.
  - Conversions – for scheelite, 1 t scheelite = 0.638 t W or 1 t W = 1.566 t wolfram.
- Tantalum (Ta) – contained Ta in metallic form, oxides, concentrates (e.g., tantalite concentrate) or direct shipping ore.
  - *Mass Units* – dry metric tonnes (adjusted for moisture content if reported as wet tonnes).
  - *Grade Units* – all converted to percent elements (%Ta), grams per metric tonne (g/t Ta) or most commonly as oxides (%Ta_2_O_5_, g/t Ta_2_O_5_).
  - Conversions – for scheelite, 1 t scheelite = 0.638 t W or 1 t W = 1.566 t wolfram.
- Niobium (Nb) – contained Nb in metallic form, oxides, concentrates (e.g., tantalite-columbite concentrate, containing both Nb and Ta) or direct shipping ore.
  - *Mass Units* – dry metric tonnes (adjusted for moisture content if reported as wet tonnes).
  - *Grade Units* – all converted to percent elements (%Nb), grams per metric tonne (g/t Nb) or most commonly as oxides (%Nb_2_O_5_, g/t Nb_2_O_5_).
  - Conversions – assuming ferro-columbite (FeNb_2_O_6_), 1 t columbite = 0.550 t Nb or 1 t Nb = 1.817 t columbite.
- Cadmium (Cd) – only extracted at Pb smelters or Zn refineries, either in oxide or refined metal forms.
  - *Mass Units* – dry metric tonnes.
  - *Grade Units* – all converted to percent elements (%Cd), grams per metric tonne (g/t Cd).
- Heavy Mineral Sands (HMS) – a body of minerals which are considerably more dense (i.e., heavier) than silica and form deposits with a variety of minerals, including rutile (TiO_2_), leucoxene (rutile with some impurities), ilmenite (broadly given as FeTiO_3_), zircon (ZrSiO_4_), rare earth minerals (monazite, xenotime), garnet, staurolite and others.
  - *Mass Units* – dry metric tonnes.
  - *Grade Units* – all converted to percent minerals (%HMS) or individual minerals (e.g., %rutile) or constituent oxides (e.g., %TiO_2_).
- Rutile – titanium oxide (TiO_2_) mineral extracted during mining for heavy mineral sands, typically left as an oxide or mineral concentrate (as titania, TiO_2_).
  - *Mass Units* – dry metric tonnes.
  - *Grade Units* – all converted to percent mineral (%rutile) or contained titania (TiO_2_).
  - Conversions – rutile concentrates are typically 95% TiO_2_.
- Leucoxene – an impure titanium oxide (TiO_2_) mineral extracted during mining for heavy mineral sands, typically left as an oxide or mineral concentrate (as titania, TiO_2_).
  - *Mass Units* – dry metric tonnes.
  - *Grade Units* – all converted to percent mineral (%leucoxene) or contained titania (TiO_2_).
  - Conversions – leucoxene concentrates are typically 70–90% TiO_2_.
- Ilmenite – an iron titanium oxide (FeTiO_3_) mineral extracted during mining for heavy mineral sands, typically left as an oxide or mineral concentrate (as titania, TiO_2_).
  - *Mass Units* – dry metric tonnes.
  - *Grade Units* – all converted to percent mineral (%ilmenite) or contained titania (TiO_2_).
  - Conversions – ilmenite concentrates are typically 30–60% TiO_2_.
- Synthetic Rutile – this is ilmenite which has been smelted to produce a rutile-like product with the same TiO_2_ content as normal rutile.
  - *Mass Units* – dry metric tonnes.
  - *Grade Units* – all converted to percent mineral (%rutile) or contained titania (TiO_2_).
  - Conversions – synthetic rutile concentrates are typically 92% TiO_2_.
- Zircon – this is zirconium silicate (ZrSiO_4_) extracted during mining for heavy mineral sands, typically left as an oxide or mineral concentrate.
  - *Mass Units* – dry metric tonnes.
  - *Grade Units* – all converted to percent mineral (%zircon) or contained zirconia (ZrO_2_).
  - Conversions – zircon concentrates are typically 65% ZrO_2_, where 1 t ZrO_2_ = 0.740 t Zr or 1 t Zr = 1.351 t ZrO_2_.
- Monazite – this is a rare earth phosphate mineral, where there is widespread substitution between individual rare earth elements, uranium and thorium in the mineral. Monazite is typically extracted from heavy mineral sands concentrates but can also be found as a host mineral in primary rare earth element deposits. The extent of elemental substitutions can vary considerably between different deposits, fields and regions of the world.
  - *Mass Units* – dry metric tonnes.
  - *Grade Units* – all converted to percent mineral (%monazite), contained rare earth oxides (%REO) or sometimes contained thorium oxides (%ThO_2_).
  - Conversions – see notes on *Rare Earth Elements*.
- Xenotime – an yttrium phosphate mineral, which can include modest substitution between yttrium and other rare earth elements, uranium and thorium in the mineral. Xenotime is typically extracted from heavy mineral sands concentrates but can also be found as a host mineral in primary rare earth element deposits. The extent of elemental substitutions can vary considerably between different deposits, fields and regions of the world.
  - *Mass Units* – dry metric tonnes.
  - *Grade Units* – all converted to percent mineral (%xenotime), contained rare earth oxides (%REO) or sometimes contained thorium oxides (%ThO_2_).
  - Conversions – see notes on *Rare Earth Elements*.
- Bismuth (Bi) – typically only extracted at Cu smelters or in concentrates (especially Cu).
  - *Mass Units* – dry metric tonnes.
  - *Grade Units* – all converted to percent elements (%Bi), grams per metric tonne (g/t).

## Data Records

### Mine by mine

The complete data sets^64^ are available from the RMIT Figshare platform (https://rmit.figshare.com/), with the citation being:

Mudd, Gavin (2023): A Comprehensive Dataset for Australian Mine Production 1799 to 2021. RMIT University figshare. Dataset. 10.25439/rmt.22724081.v2^64^.

The principal files available are:

- Principal File (Excel or xlsx format): “Aust-Mine-Prod-v01.4b-Master.xlsx”, note this includes multiple tabs (explained below).
  - *‘Notes’ tab* – descriptive information for the data set, including key assumptions, references, acronyms and symbols.
  - *‘Summary’ tab* – the cumulative production data for mines and fields compared with reported time series production for each element (or minerals in some cases).
  - *‘Mine by Mine’ tab* – the detailed cumulative production data for all individual mines and fields for each element (or minerals in some cases).
  - *‘Annual Data’ tab* – the reported annual time series production data by state and national levels for each element (or minerals in some cases).
  - *‘Sn Fields’ tab* – the reported annual time series production data by each individual tin mining field in each state or territory around Australia.
- Comma Separated Values (CSV format):
  - Aust-Mine-Prod-v01.4b-Tab1-Notes.csv
  - Aust-Mine-Prod-v01.4b-Tab2-Summary.csv
  - Aust-Mine-Prod-v01.4b-Tab3-Mine-by-Mine.csv
  - Aust-Mine-Prod-v01.4b-Tab4-Annual-Data.csv
  - Aust-Mine-Prod-v01.4b-Tab5-Sn-Fields.csv
- Graphs of Mine Production versus Reported Production (PowerPoint or pptx format):
  - Aust-Mine-Prod-v01.4b-Tab06-11-Graphs.pptx

The primary variables included in this data set are:

- Mine/Project/Field (column A in Excel file) – simply the most common name for each mine, mining project or mining field. For some sites, names can change over time, sometimes frequently, so the name as at 2021 is typically chosen (earlier names are often stated in the ‘Notes’ balloon for that cell).
- State or Territory (column B in Excel file) – simply.
- Operating Period (column C in Excel file) – this is the years for which the site has been operated (e.g., years 1938 to 1962), noting that activities may not be continuous during the period stated. It should also be noted that at some sites not all years of operations will have data, meaning the cumulative site shown remains incomplete (this is a very small number of sites).
  - *Discontinuous Operating* – sometimes a mine may go into care and maintenance or shut down but is later reopened due to changing market conditions, exploration success or new owners, noted with an ‘@’ symbol (e.g., 1894–2014^@^).
  - *Still Operating* – if a mine was still in operation at the end of 2021 (the end year for the current data set), it is noted with a hash (e.g., 1988–2021^#^).

- Ore Processed (column D in the Excel file) – stated in million tonnes (Mt), 1 Mt is equivalent to 1 Tg or 10^12^ g, this is the ore which has been processed in some manner to extract a metal, mineral or other saleable product. Although this is very commonly and widely reported by mines, there are some where no data is reported or available for ore processed despite extracted products being reported (e.g., for the Greenbushes mine, ore processed to extract tin, lithium and/or tantalum products is rarely report although considerable data is available on extracted products).
- Ore Grades (columns E to Q in the Excel file) – this represents the quantity or concentration of a metal or mineral in the ore processed. For the 1850s, this typically means yield only – that is, 1 tonne of ore processed leads to production of 31.1 g (or 1 ounce) of gold – a yield of 31.1 g/t Au. It is important to remember that not all of the contained metal in ore is extracted during processing, leaving some behind in tailings (see later definition). From about 1900, assaying of ore became more common across the mining sector, which means ore grades represent the total quantity of a metal or mineral in the ore being processed. There are grade variables for all of the most common metals or minerals (columns D to N), with ‘other’ (columns O to P) allowing for uncommon commodities to be stated as needed (e.g., antimony, uranium oxide, etc).
- Direct Shipping Ore (DSO) (columns R to Y in the Excel file) – this is relatively rich or high grade ore which is not processed on site and is simply excavated and shipped to a customer. Examples include gold ore shipped to a smelter or refinery, oxide ores shipped to a smelter or iron ore of sufficient grade and quality to be sold directly, amongst others. DSO does not leave any tailings behind.
- Concentrate (columns Z to AX in the Excel file) – this is a metal-rich product extracted during ore processing at a mine, most commonly using flotation technology (a process using water-oil mixtures and bubbling air through finely ground ore to separate sulfide minerals from the non-economic minerals). Typically, a concentrate is produced for a primary metal (e.g., copper, lead, zinc, nickel or tin) but may also include other metals of economic value (e.g., gold, silver, platinum group elements, other metals).
  - *Copper concentrates* (columns Z to AE in the Excel file) – minor to significant gold and silver, with sometimes important amounts of lead or zinc.
  - *Lead concentrates* (columns AF to AK in the Excel file) – modest to significant silver, with sometimes important amounts of zinc, copper, gold or sub-economic amounts of antimony, cadmium, manganese, etc.
  - *Zinc concentrates* (columns AL to AQ in the Excel file) – modest silver, with sometimes important amounts of lead, copper, gold or sub-economic amounts of cobalt, cadmium, manganese, etc.
  - *Nickel concentrates* (columns AR to AX in the Excel file) – modest copper, minor cobalt, sometimes important amounts of platinum group elements, gold or silver.

- Tin Oxide (columns AY to BB in the Excel file) – tin is often widely extracted as the oxide mineral cassiterite (SnO_2_) from alluvial geological systems, either by prospectors, sluicing or dredging methods or in many cases by hard rock mines often referred to as lode mines. For many fields producing tin, it is clear that tin was extracted from alluvial sources (e.g., prospectors, sluicing, dredging), but for some fields both lode and alluvial mining occurred – tin mining has been allocated to either alluvial or lode based on the best available data, noting that some fields there is no distinction (in which case it is assumed to be alluvial). At some mines, tin is extracted from sulfide-based ores (e.g., stannite), with this data being placed in the lode tin column.
- Tungsten: Wolfram and Scheelite (columns BC to BF in the Excel file) – tungsten is typically found in the minerals wolfram or scheelite and can be mined using alluvial or hard rock methods. Given the dominance of one mineral over another, the production is stated as wolfram or scheelite concentrate with the tungstic trioxide content (%WO_3_). Cumulative production data are stated as contained tungstic trioxide (t WO_3_).
- Iron Ore (columns BG to BH in the Excel file) – known as saleable iron ore (or concentrate), generally with low impurities. Cumulative production is stated as contained lithium (t Fe ore) with concentrate grade (%Fe).
- Lithium (Spodumene) (columns BI to BJ in the Excel file) – the most common economic lithium mineral is spodumene (lithium silicate), with data being stated as tonnes spodumene plus contained lithia (Li_2_O). Cumulative production is stated as contained lithium (t Li).
- Other Concentrates (columns BK to BR in the Excel file) – this is commonly metal-rich concentrates which are not solely considered a dominant metal but instead are sold containing mixtures of common metals. For example, some concentrates are sold as copper-lead or lead-zinc, a metal-containing pyrite concentrate, or may refer to another metal or mineral entirely, such as:
  - *Base metal concentrates* – includes a variable fraction of copper, lead and/or zinc plus useful gold and/or silver (e.g., Broken Hill, Hellyer, Golden Grove, Hera, Peak, CSA, Captain’s Flat, McArthur River, Thalanga).
  - *Gold concentrates* – in rare circumstances, a gold-dominant concentrate can be produced with variable silver but low base metals (e.g., Mineral Hill, Mount Carlton).
  - *Silver concentrates* – in rare circumstances, a silver-dominant concentrate can be produced with variable gold and/or copper, lead (e.g., Mount Carlton).
  - *Iron concentrates* – sites which produce a saleable iron ore product but containing other metals of potential economic value such as copper (e.g., Cairn Hill).
  - *Antimony concentrates* – antimony sulfide (stibnite) concentrate, typically containing significant gold (although the recoverability of this gold is complex and variable).
  - *Pyrite concentrates* – pyrite is iron sulfide (FeS_2_) and can be used to make sulfuric acid; at some mines a pyrite concentrate was extracted and sold (e.g., Mount Lyell, Rosebery-Hercules) or was the sole product (e.g., Brukunga).
  - *Precipitates concentrates* – these are precipitates produced from the treatment of acid mine waters, typically copper with minor gold and/or silver (e.g., Clonclurry field, Mount Perry, Mount Cannindah). The term cement can also be used in place of precipitates.
  - *Diamonds* – these are typically extracted as pure minerals or diamonds.
  - *Gravity concentrates* – gold mines often incorporate a circuit to capture coarse gold through gravity techniques, although this data is rarely reported separate (gravity gold concentrates can be up to 50% Au). Gravity concentrates can also be produced for other metals minerals.
  - *Manganese concentrates* – manganese dioxide or pyrolusite (MnO_2_) is widely used as a chemical reagent, in certain steel alloys or as a component for energy storage batteries. At mines, it is typically extracted from ore processing to produce a pyrolusite concentrate, often referred to as manganese ore (especially if it is sufficiently high grade to be classified as direct shipping ore) or manganese concentrate.
  - *Chromite concentrates* – chromite is an oxide mineral containing iron, magnesium and chromium ((Fe,Mg)Cr_2_O_4_). Ore containing chromite is processed to produce a chromite-rich concentrate, with grades typically given as %Cr_2_O_3_ (some ore may be considered sufficiently rich to be direct shipping ore).
  - *Phosphate Rock concentrates* – ore containing high concentrations of phosphate (PO_4_) can be directly shiiped, although more commonly ore is simply processed to produce a phosphate-rich concentrate, commonly referred to as phosphate rock (e.g., Christmas Island), or chemically processed to specific phosphate products (e.g., Phosphate Hill).
  - *Brown Coal* – stated as brown coal, effectively a direct shipping ore.
  - *Alumina concentrates* – the processing of bauxite ore gives a high purity alumina product (which is subsequently used in a smelter to produce aluminium). Typically only tonnes alumina is stated with no grade provided.
  - *Other concentrates* – this includes concentrates for uncommon metals such as molybdenum, bismuth or tantalum (as tantalite, Ta_2_O_5_).

- Metals or Minerals Extracted (columns BS to CM in the Excel file) – this represents mine production for the range of metals or minerals extracted at that site or field. In general, most data are presented in elemental form (e.g., Au, Cu, Ni), whilst some are left in mineral form as this is how they are sold (e.g., Mn concentrate, chromite, bauxite, alumina, etc).
- Tailings-Derived Gold (column CN in the Excel file) – due to the historical reprocessing of gold tailings, it is possible for many older fields to distinguish production between fresh ore and tailings. This value represents all gold derived from the reprocessing of old gold tailings – effectively meaning that the yields as shown for the various fields would be a bit higher to reflect the residual gold in tailings. Caution should be applied, however, as the extent of tailings reprocessed is not always well documented (especially in the latter 1800s, after which time government statistical surveys generally capture such activity).
- Dollied Gold (column CO in the Excel file) – a particular type of individual prospecting only reported on in Western Australia. Dollying is the process of using a gold pan and dolly pot to isolate gold nuggets found in alluvial soils.
- Alluvial Gold (column CP in the Excel file) – this is general gold found by individual prospectors in the surface environment (or regolith) with no documented method.
- Waste Rock (column CQ in the Excel file) – rock mined which has uneconomic concentrations of metals or minerals and is treated as solid waste. Waste rock is typically only partially reported in modern mines, hence much remains as best estimates (which may often still be missing many years of data), with no systematic data for historic fields. Waste rock quantities are substantial for open pit mines (e.g., up to several fold of the ore processed or even higher in some cases) but typically a fraction of ore processed for underground mines.
- Metals/Minerals Extracted Estimated (column CR in the Excel file) – this is whether the specific metals or minerals noted are estimated data (i.e., includes some calculated or assumed data).
- Waste Rock Estimated (column CS in the Excel file) – this is whether the waste rock (aka overburden) is estimated data (i.e., includes some calculated or assumed data).
- Status (column CU in the Excel file) – whether a mine is operating (Op), in care and maintenance (C&M), closed and undergoing rehabilitation (Closed), finished rehabilitated (Rehab) or abandoned (Aban).
- Metals/Minerals (column CV in the Excel file) – the specific metals or minerals which have been extracted by that mine.

### Annual data

The primary variables included in this data set are a detailed time series of the metals or minerals produced by mining for each state and nationally. For example, total mined gold production is given from 1851 to 2021 for each state and territory. The synthesized time series for all metals and minerals only show states for which production is reported or known. The time series give rise to cumulative totals by state and nationally. Furthermore, sub-totals are given for certain periods to allow comparison of changes in mining over time; specifically, cumulative production is shown to the year 1900, 1901 to 1950, 1951 to 2000 and 2001 to 2021 – loosely aligned with key changes in technology, scale, new discoveries and economics of mining across Australia (as well as other interruptions such as world wars, long workers strikes, etc).

### Tin by field (‘Sn Field’)

As an example, the more detailed time series for tin production by principal field across Australia is also included. This allows the tin to be placed geographically to its origin. Such data also allows the dominant regions for tin production to be clearly identified, such as the Herberton-Chillagoe and Stanthorpe regions of Queensland, north-east Tasmania, the Peel-Uralla and New England regions of north-east New South Wales, or larger mines such as Mount Bischoff, Renison Bell and Greenbushes. Future extensions of this data set are planned to add in field by field or mine-by-mine data for numerous other commodities (e.g., gold, copper, lead, zinc, iron ore, etc).

## Technical Validation

The data presented in this compilation assume that the principal sources of government statistical series and reports and mining company reporting have their own quality control. This is accepted practice for such official data and is consistent with the older Australian study by Kalix *et al*.^19^ as well as numerous other national such studies (e.g., Canada, United States of America, United Kingdom, Germany, etc).

To ascertain the extent to which cumulative mine production data cover a particular metal or mineral, the totals of mine production at a national level are compared with the cumulative production from the national time series data. For example, mined gold production totals 15,101 t Au compared to national production of 15,304 t Au – showing that the data synthesized herein covers 98.7% of reported gold production across Australia. This demonstrates that the mine data are indeed a most robust coverage of the Australian gold mining sector and its production. Similar results are shown for most metals and minerals, with the overall results shown below in Tables 1–4. Table 5 shows the cumulative production by major periods of Australia mining history.

**Table 1:** Summary Data for Individual Mines – Precious Metals and Diamonds.

	Gold (t Au)	Silver (t Ag)	Platinum Group Elements (t PGEs)	Diamonds (Mcarats)
Number of Producing Mines	340	137	5	5
Queensland	1,633.1	47,240	—	—
New South Wales	1,151.4	43,039	0.660	—
Victoria	2,659.1	—	0.967	—
Tasmania	245.7	8,236.8	0.0097	—
South Australia	186.0	1,135.1	—	—
Western Australia	8,542.8	2,224.2	29.2	918.0
Northern Territory	682.9	1,593.7	—	0.529
Australian Territories	—	—	—	—
Australia: Mines & Fields	15,101	103,469	30.8	918.5
Australia: Cumulative	15,304	98,664	31.6	918.5
Ratio (%) (mines/cumulative)	**98.7%**	**104.9%**	**97.6%**	**100.0%**

**Table 2:** Summary Data for Individual Mines – Base Metals.

	Copper (kt Cu)	Lead (kt Pb)	Zinc (kt Zn)	Nickel (kt Ni)	Cobalt (kt Co)	Tin (kt Sn)
Number of Producing Mines	147	47	33	25	11	41
Queensland	14,416	15,492	22,421	327.4	14.6	191.6
New South Wales	5,370.5	25,628	27,006	—	0.96	178.0
Victoria	—	—	—	—	—	10.7
Tasmania	2,310.1	2,524.8	6,380.6	3.73	—	471.5
South Australia	6,572.2	68.2	367.0	—	—	—
Western Australia	3,232.7	1,117.0	4,215.6	5,784.8	87.3	37.1
Northern Territory	410.1	1,219.1	5,256.4	—	—	2.93
Australian Territories	—	—	—	—	—	—
Australia: Mines & Fields	32,311	46,049	65,647	6,116.0	102.9	891.8
Australia: Cumulative	32,258	45,098	65,510	6,108.6	141.6	893.7
Ratio (%) (mines/cumulative)	**100.2%**	**102.1%**	**100.2%**	**100.1%**	**72.6%**	**99.8%**

**Table 3:** Summary Data for Individual Mines – Iron Ore, Steel Alloy Metals and Bauxite-Alumina.

	Iron Ore (Mt Fe ore)	Manganese (Mt Mn conc)	Chromium (kt Cr conc)	Molybdenum (kt Mo)	Bauxite (Mt bauxite)	Alumina (Mt alumina)
Number of Producing Mines	45	3	1	2	14	8
Queensland	—	—	—	0.413	780.1	197.8
New South Wales	—	—	—	—	—	—
Victoria	—	—	—	—	—	—
Tasmania	115.1	—	—	0.231	—	0.414
South Australia	374.9	—	—	—	—	—
Western Australia	13,821	16.0	3,115.2	—	1,488.4	430.8
Northern Territory	25.7	141.7	—		318.3	63.4
Australian Territories	—	—	—	—	—	—
Australia: Mines & Fields	14,337	157.7	3,115.2	0.644	2,579.5	651.4
Australia: Cumulative	14,837	158.8	3,193.2	1.82	2,5886.8	692.3
Ratio (%) (mines/cumulative)	**96.6%**	**99.3%**	**97.6%**	**35.5%**	**100.3**	**94.1**

**Table 4:** Summary Data for Individual Mines – Specialty Metals and Uranium.

	Lithium (kt Li)	Tungsten (kt WO_3_)	Antimony (kt Sb)	Rare Earth Oxides (kt REO)	Cadmium (kt Cd)	Uranium (kt U_3_O_8_)
Number of Producing Mines	5	20	2	1	1	14
Queensland	—	15.4	—	—	—	8.89
New South Wales	—	—	19.9	—	—	—
Victoria	—	0.058	54.8	—	—	—
Tasmania	—	60.1	0.228	—	3.07	—
South Australia	—	0.0023	350.7	—	—	122.3
Western Australia	212.0	—	—	190.7	—	0
Northern Territory	—	0.873	—	—	—	147.5
Australian Territories	—	—	—	—	—	—
Australia: Mines & Fields	212.0	76.4	425.5	190.7	3.07	278.7
Australia: Cumulative	412.1	104.1	490.0	362.9	33.0	277.4
Ratio (%) (mines/cumulative)	**51.4%**	**73.4%**	**86.8%**	**52.5**	**9.3**	**100.5%**

**Table 5:** Cumulative Australian mine production by major time period and metal and mineral.

Element/Mineral	Units & Symbol	by 1900	1901–1950	1951–2000	2001–2021	Cumulative
Gold	t Au	2,831	2,401	4,393	5,679	15,304
Silver	t Ag	5,400	16,849	40,427	35,988	98,664
Copper	kt Cu	521.2	1,243	11,559	18,936	32,258
Lead	kt Pb	1,130	9,748	21,302	12,918	45,098
Zinc	kt Zn	50.6	7,375	29,688	28,396	65,510
Nickel	kt Ni	0	0.65	2,312	3,796	6,108
Cobalt	kt Co	0.035	1.07	34.2	106.3	141.6
Iron Ore	Mt Fe ore	0.082	49.5	3,417	11,370	14,837
Tin	kt Sn	208.8	217.1	348.7	119.0	893.7
Tungsten	kt WO_3_	0.62	23.5	77.9	2.10	104.1
Uranium	kt U_3_O_8_	0	0.028	107.5	169.9	277.4
Manganese	Mt Mn conc	0.016	0.15	55.6	103.1	158.9
Black Coal	Mt raw	102.0	577.9	5,290	10,072	16,042
Brown Coal	Mt	0.055	99.4	1,565	1,273	2,938
Platinum Group	t PGEs	0.21	1.22	14.9	15.2	31.6
Antimony	kt Sb	18.7	66.6	345.6	59.2	490.0
Phosphate Rock	Mt PO_4_ rock	0.15	6.07	45.2	43.3	94.6
Diamonds	Mcarats	0.090	0.11	571.4	346.9	918.5
Rare Earth Oxides	kt REO	0	0.20	154.8	207.9	362.9
Gallium	t Ga	0	0	85.4	0	85.4
Bauxite	Mt	0	0.071	1,008	1,572	2,580
Alumina	Mt	0	0	281.8	410.6	692.3
Vanadium	kt V	0	0	2.86	11.4	14.3
Chromium	kt Cr	12.4	14.5	144.7	1,482	1,654
Graphite	kt	0.64	5.01	1.15	0.020	6.81
Lithium	kt Li	0	0.0010	43.6	368.4	412.0
Molybdenum	kt Mo	0.0056	1.45	0.34	0.017	1.82
Tantalum	t Ta	0	0.29	6.94	5.57	12.8
Niobium	t Nb	0	0.0016	0.00009	0	0.0017
Cadmium	kt Cd	0	5.80	25.2	2.01	33.0
Garnet	kt garnet	0	0.0091	875.5	5,859	6,735
Ilmenite	kt ilmenite	0	18.3	38,331	16,354	54,703
Rutile	kt rutile	0	129.4	10,094	4,861	15,084
Leucoxene	kt leucoxene	0	0	580.4	796.7	1,377
Zircon	kt zircon	0	185.5	15,783	10,857	26,825
Monazite	kt monazite	0	0.33	258.8	27.0	286.1

As shown in Tables 1–4, for most commodities summed or cumulative individual mine production represents some 95% to ~100% of cumulative production (e.g., Au, Fe ore, Sn, U, Pb, Zn, Ag, Mn, Diamonds, brown coal, platinum group elements, phosphate rock, bauxite-alumina, chromium), demonstrating that the individual mine data is a clear and comprehensive coverage of these Australian mining sectors. For other sectors, however, the coverage is not as comprehensive and ranges from 9.3% to 86.8% (e.g., REOs, Cd, Mo, Bi, Co, Li), data remains to be compiled and synthesized in the same manner (e.g., black coal, heavy mineral sands, graphite, garnet, Ta, V), major mines are still missing (e.g., Li and Greenbushes) or was produced mostly by prospectors or prospecting syndicates (e.g., Nb, W, noting that considerable Sn production was through prospecting and syndicates but this is captured within the field data included).

For Table 5, the two most important observations are the increasing diversity of metals and minerals produced over time and the rapidly increasing scales of production across most commodities. For diversity, it can be seen that 17 of the 36 metals and minerals show no production by 1900, with a further 13 having very minimal production in comparison to say the 2001 to 2021 period, leaving just 6 principal metals and minerals by 1900 (gold, tin, black coal, copper, silver and tin). For scale, different metals or minerals highlight different issues. Gold, for example, shows nearly twice as much production from 2001 to 2021 as that from 1851 to 1900 – driven by new process technology, favourable economics (especially the long-term increase in the real price of gold) as well as ongoing success in exploration finding new deposits or extensions to known deposits. In contrast, iron ore shows an extra-ordinary increase in production since 1950 – driven by favourable markets and ongoing exploration success. For other metals or minerals, they reflect the ever-increasing demand for a wider array of metals and minerals to underpin material consumption, materials and modern technologies (e.g., the rise and now gradual fall of uranium, the emergence of heavy mineral sands, rare earths, lithium, etc).

## Usage Notes

The data synthesized in this compilation are for researchers, policy analysts, economic modellers, environmental advocates and anyone to use to help with their efforts and work. The data is provided as is within the spreadsheet and can be tabulated in different ways, plotted as time series graphs or converted to financial value for comparisons. The data could even be useful to inform modelling of various sorts, such as econometric modelling, life cycle assessment modelling, biodiversity impacts, carbon pollution estimates and the like. All usage of this data should acknowledge and formally cite this publication as the source in the normal academic manner.

Some important notes concerning estimated and assumed values for some mines and/or years, plus a brief discussion of mine tailings:

- Estimated Values – sometimes actual values are not reported but there is sufficient information and related data which justify an approximate value being derived. For example, a mining project has been operating for several years but does not state ore processed for that year – given other nearby years (before and after) and typical recovery rates and ore grades, it is possible to derive an estimate of ore processed for the missing year. In general, this approach works well and gives a reasonable basis in the absence of the specific value being reported. For this data set, any such values where there is more than 20% of the cumulative data is estimated, values are given in bold-blue text to show they include some estimated vales.
- Assumed Values – for some mines or mining fields, there are no data reported on aspects such as ore grades and ore processed – although there will be extracted metals or minerals. For such cases, it is possible to assume typical ore grades (based on other fields of the day or typical values) and then estimate ore processed. If values are assumed, they are stated in bold-red text.
  - *Example: Gold mining* – gold mines and fields of the 1850s in Victoria did not report ore processed until about 1859^26^. We know that most gold produced during this time was by prospectors, so we can use the available data from 1859 onwards (which includes ore processed, yields and production) to assume typical ore grades and then extrapolate the quantity of gold produced by hard rock versus prospectors, assume typical ore grades or yields, and then estimate ore processed. Given the lack of historical data, this represents the best approach possible and gives a reasonable order of magnitude to the scale of gold mining for the 1850s.

- Tailings – after ore processing and the extraction of a particular product (e.g., metal, concentrate, saleable ore), the residual minerals are referred to as tailings. These are typically fine-grained in texture and may still contain some metals or minerals of potential economic interest. Tailings historically used to be discharged to streams or adjacent land in the 1850s, with the use of engineered storage dams from about 1900 or so. In some sectors, it has been common to go back and reprocess old tailings to extract additional metals or minerals (particularly in the gold sector). No estimates of tailings are provided specifically in this data set, but where possible, some individual tailings reprocessing projects are included (e.g., Broken Hill, Kaltails). At some mines, historic tailings have been reprocessed but production data covered both fresh ore as well as tailings, thereby precluding separate production data for primary ore versus tailings (e.g., Herberton tin field, Woodlawn, Greenbushes).

## Supplementary information

A Comprehensive dataset for Australian mine production 1799 to 2021

## Data Availability

No custom code was developed for this work and data set.
