# Supplementary material for: A Comprehensive dataset for Australian mine production 1799 to 2021
Source: Sci Data. 2023 Jun 20;10:391. doi: 10.1038/s41597-023-02275-z (PMC10281968; doi:10.1038/s41597-023-02275-z)
Supplement: Supplementary file 1 — A Comprehensive dataset for Australian mine production 1799 to 2021 [file 41597_2023_2275_MOESM1_ESM.docx]

**A Comprehensive Dataset for Australian Mine Production, 1799 to 2021**

*Supplementary Information*

Gavin M. Mudd^1^

^1^Environmental Engineering, School of Engineering, RMIT University, 124 La Trobe Street, Melbourne, VIC, Australia 3000 (**Gavin.Mudd@rmit.edu.au**)

1. **Departmental Websites Consulted (Over a long history of ~20 years or so):**

Government departments are often merged, split, rearranged and/or renamed over time, making the list below an approximate one only. The website for the current name is provided. Note that until about the 1970s, all state departments were typically still called the ‘Department of Mines’. No dates have been set for the names of each title as this is beyond the scope of the paper but also does not limit the ability to find any specific source as the current name and website can be used to contact the relevant part of the Department or find the statistical reports and source used.

*All States (historical):*

**Department of Mines** – up to the 1980s or so (depending on each state’s changes to bureaucratic structure).

*Tasmania (current):*

Mineral Resources Tasmania, Department of Primary Industries, Parks, Water & the Environment

<https://www.mrt.tas.gov.au/>

*Tasmania (previous):*

Mineral Resources Tasmania, Department of Infrastructure, Energy & Resources

Mineral Resources Tasmania, Department of Development & Resources

Department of Mines

*Victoria (current):*

Earth Resources Regulation (within Department of Energy, Environment & Climate Action)

<https://earthresources.vic.gov.au/>

*Victoria (previous):*

Department of Minerals & Petroleum

Office of Minerals & Energy

Department of Minerals & Energy

Department of Mines

*New South Wales (current):*

Mining, Exploration & Geoscience (MEG), Department of Regional NSW

<https://meg.resourcesregulator.nsw.gov.au/>

*New South Wales (previous):*

Department of Primary Industries

Resources & Energy, Department of Industry

Department of Mineral Resources

Department of Mines & Energy

Department of Mines

*Queensland (current):*

Mining & Exploration, Department of Resources

<https://www.resources.qld.gov.au/mining-exploration>

*Queensland (previous):*

Department of Natural Resources & Mines

Mines & Energy, Department of Employment, Economic Development & Innovation

Department of Mines & Energy

Department of Resource Industries

Department of Mines

*South Australia (current):*

Department for Energy & Mining

<https://www.energymining.sa.gov.au/>

*South Australia (previous):*

Resources & Energy, Department of State Development

Primary Industries & Resources South Australia

Department of Mines & Energy

Department of Mines

*Western Australia (current):*

Department of Mines, Industry Regulation & Safety

<https://www.dmirs.wa.gov.au/>

*Western Australia (previous):*

Department of Mines & Petroleum

Department of Industry & Resources

Department of Minerals & Energy

Department of Mines

*Northern Territory (current):*

Mining & Energy, Department of Industry, Tourism & Trade

<https://nt.gov.au/industry/mining-and-energy>

*Northern Territory (previous):*

Department of Resources

Department of Primary Industry & Resources

Department of Regional Development, Primary Industry, Fisheries & Resources

Department of Primary Industry, Fisheries & Mines

Department of Business, Industry & Resource Development

Department of Mines & Energy

Department of Mines

*Australian Government (current):*

Department of Industry, Science & Resources

<https://www.industry.gov.au/>

Office of the Chief Economist

<https://www.industry.gov.au/office-chief-economist>

Geoscience Australia

<https://www.ga.gov.au/>

Australian Bureau of Agricultural, Resource Economics & Science, Department of Agriculture, Fisheries & Forestry

<https://www.agriculture.gov.au/abares>

*Australian Government (previous):*

Department of Industry, Tourism & Resources

Department of Resources, Energy & Tourism

Department of Resources

Australian Bureau of Agricultural & Resource Economics

Australian Geological Survey Organisation

Department of Northern Australia

Bureau of Mineral Resources, Geology & Geophysics

1. **Corporate & Related Websites Consulted (Over a long history of ~20 years or so):**

Since the emergence of the internet, companies now have the opportunity to publish and make available their own financial reports, media releases, technical studies and project information on their own websites. Furthermore, regulators also make available reports and documents through their own website. The list of regulator websites, corporate information database services as well as professional societies or international organisations with relevant publications and data include:

*Australian Securities Exchange (ASX):*

<https://www2.asx.com.au/>

MorningStar DataAnalysis – historical archive of ASX releases

<https://datanalysis.morningstar.com.au/> (note: institutional subscription or account required)

*Australasian Institute of Mining & Metallurgy (AusIMM):*

<https://www.ausimm.com/>

*United States Geological Survey (USGS):*

<https://minerals.usgs.gov/>

*The Internet Archive:*

<https://archive.org/>
